# Systematic review of search for meaning in life assessment tools: highlighting the need for a quest for significance scale

**DOI:** 10.3389/fpsyg.2025.1513720

**Published:** 2025-03-24

**Authors:** Fahd Alsaadi, Miguel A. Maldonado, Mohammad Erfanikia, Erica Molinario, Manuel Moyano

**Affiliations:** 1 Departamento de Psicología, Facultad de Ciencias de la Educación y Psicología, Universidad de Córdoba, Córdoba, Spain; 2 Hospital Universitario “Reina Sofía”, Instituto Maimónides de Investigación Biomédica de Córdoba, Córdoba, Spain; 3 School of Psychology, University of East Anglia, Norwich, United Kingdom; 4 Department of Psychology, Florida Gulf Coast University, Fort Myers, FL, United States; 5 Institute of Psychology, Jagiellonian University, Kraków, Poland

**Keywords:** quest for significance, meaning in life, search for meaning, psychometric assessment, bibliometric analysis, systematic review

## Abstract

**Objective:**

This systematic review aims to evaluate the adequacy of existing assessment tools in measuring the search for meaning in life and the quest for significance, with a focus on identifying gaps in capturing the quest for significance as a distinct construct.

**Methodology:**

Following the PRISMA protocol, we conducted a comprehensive search across ProQuest, Web of Science, and Scopus, identifying 23 relevant studies. Methodological quality was assessed using the Downs and Black checklist and the Strengthening the Reporting of Observational Studies in Epidemiology (STROBE) checklist. A bibliometric analysis was also performed to explore research trends and collaboration patterns.

**Key findings:**

While numerous instruments exist, none fully address the quest for significance as a standalone construct. Key limitations include insufficient differentiation between personal and social significance, lack of predictive validity, and limited cultural adaptability.

**Conclusion and implications:**

The findings underscore the need for a dedicated Quest for Significance Scale to advance psychological research and practice. Future efforts should focus on developing robust, culturally adaptable instruments to better understand the quest for significance across diverse contexts.

## 1 Introduction

The search for meaning in life is a central theme in existential psychology, reflecting individuals’ efforts to understand their purpose, coherence, and significance in the world. While meaning in life is widely recognized as comprising three dimensions, coherence (the belief that life makes sense), purpose (having direction), and significance (feeling that life has value) (Martela and Steger, 2016) the quest for significance represents a distinct but related motivational drive. Unlike the search for meaning, which focuses on understanding one’s purpose and coherence, the quest for significance emphasizes the pursuit of personal, social, and cosmic value. Despite their conceptual overlap, these constructs are not identical, and existing measurement tools often conflate or inadequately capture the quest for significance. This systematic review aims to evaluate the adequacy of current assessment methods in distinguishing between these constructs, with a focus on identifying gaps in measuring the quest for significance.

Arie Kruglanski’s Significance Quest Theory provides a foundational framework for understanding the quest for significance. According to Kruglanski, individuals are innately driven to seek personal, communal, and cosmic significance, and this drive can be dynamically activated by situational factors such as experiences of loss or gain (Kruglanski et al., 2022). The theory delineates three facets of the quest for significance—personal significance, social relevance, and cosmic significance—and highlights individual differences in how people prioritize these facets and the strategies they employ to fulfill them (Kruglanski and Orehek, 2011). This theoretical perspective underscores the importance of distinguishing the quest for significance from the search for meaning, as each has unique implications for behavior and well-being.

Empirical research supports the distinct yet interrelated nature of these constructs. For example, studies have shown that the quest for significance is positively correlated with sacrificial behaviors and extreme political attitudes, even after controlling for the search for meaning (Molinario et al., 2021). Similarly, experimental research demonstrates that instances of loss of significance can lead to a decrease in meaning in life (Stillman et al., 2009; Zadro et al., 2006). However, commonly used measures, such as the Meaning in Life Questionnaire (MLQ; Steger et al., 2006) and the Quest for Significance Scale (QFSS; Molinario et al., 2021), often fail to adequately distinguish between these constructs. While the MLQ assesses the presence of and search for meaning, and the QFSS measures the quest for significance, both tools exhibit limitations in capturing the unique motivational and behavioral outcomes associated with the quest for significance.

This systematic review seeks to address these limitations by evaluating the validity and reliability of existing assessment tools in measuring the quest for significance. Specifically, the review will: (1) determine whether current instruments implicitly or explicitly measure the quest for significance alongside the search for meaning, and (2) assess the appropriateness of these tools for capturing the quest for significance as a distinct construct. By identifying strengths, limitations, and areas for further investigation, this review aims to contribute to the development of more robust and contextually relevant assessment tools. This will advance our understanding of the quest for significance and inform research and practice in psychology, counseling, and related fields.

## 2 Methods

### 2.1 Resources and search strategy

The study selection process was carried out according to Preferred Reporting Items for Systematic Reviews and Meta-Analyses (PRISMA) guidelines to ensure a systematic and transparent approach (Page et al., 2021). The literature search was conducted between May 1, 2024, and May 31, 2024, in three sequential stages.

Initially, we conducted a search using the electronic platform ProQuest, which consolidates multiple databases such as PsycINFO, PsycARTICLES, PsycExtra, PsycTests, and Psychology Database. These databases were selected for their comprehensive coverage of psychological and social science literature, ensuring a broad and representative sample of relevant studies. Additionally, we searched two other databases: Web of Science and Scopus. We analyzed the terms that appear most frequently in basic theoretical publications and classified these keywords into two groups, which we then searched for in titles, abstracts, and keywords for study selection:

1. Instrument/Tool/Scale/Questionnaire/Assess*/Measurement;
2. “Search for Meaning”/“Quest for Significance”/“Meaning in Life”/“Significance Quest”/“Search for Significance”/“Purpose in Life”/“Search for Purpose”/“Search for Goal.”

We used the Boolean operator “AND” to couple keywords from group 1 with those from group 2. For instance, we combined Instrument* AND “Search for Meaning,” then Instrument* AND “Quest for Significance,” and so forth. The asterisk () was used to include all words starting with the same letters (e.g., Assess retrieved papers containing the words Assess, Assessment, and Assessments).

### 2.2 Eligibility criteria

Papers meeting the following criteria were included: (1) original research published in peer-reviewed journals; (2) Articles written in English, Spanish, and Arabic. These languages were selected due to the research team’s proficiency, ensuring accurate interpretation and analysis of the data. While this may limit the scope, it ensures methodological rigor in the review process. Articles requiring translation into another language were translated by bilingual team members, and translations were cross-checked for accuracy. (3) discussion of instruments that measure the search for meaning (or include this aspect as a subscale) and the quest for significance; (4) inclusion of at least some psychometrics of the discussed instrument.

Exclusion criteria encompassed adaptations of the original scale to other cultures. Cultural adaptation studies were excluded because they often focus on translating and validating existing scales rather than developing new instruments or exploring the conceptual foundations of the constructs. This exclusion ensures that our review focuses on original tools and their psychometric properties.

In our systematic review we assessed all included instruments based on the following criteria: reliability (internal consistency and retest reliability), interpretability/norms, validity (criterion and construct validity), appropriateness, feasibility, acceptability, responsiveness, and precision. Using these selection criteria, the aim of this systematic review was to comprehensively evaluate existing assessment tools that evaluate search for meaning and implicitly measure the quest for significance in life, as well as questionnaires that were developed to explicitly measure the quest for significance.

### 2.3 Study selection

The identification and selection processes were performed in a blinded manner, in which two independent reviewers used the aforementioned search strategies to first identify scientific articles only, excluding books, book chapters, conference proceedings, and reviews. Initially, we identified records from several databases, including Web of Science (WOS), Scopus, and ProQuest, yielding a total of 5,069 records. After removing 2,050 duplicates using RefWorks, 3,019 unique records remained. We then screened the titles and abstracts of these records by two independent reviewers, excluding 2,814 that did not meet the inclusion criteria.

**Figure 1:**
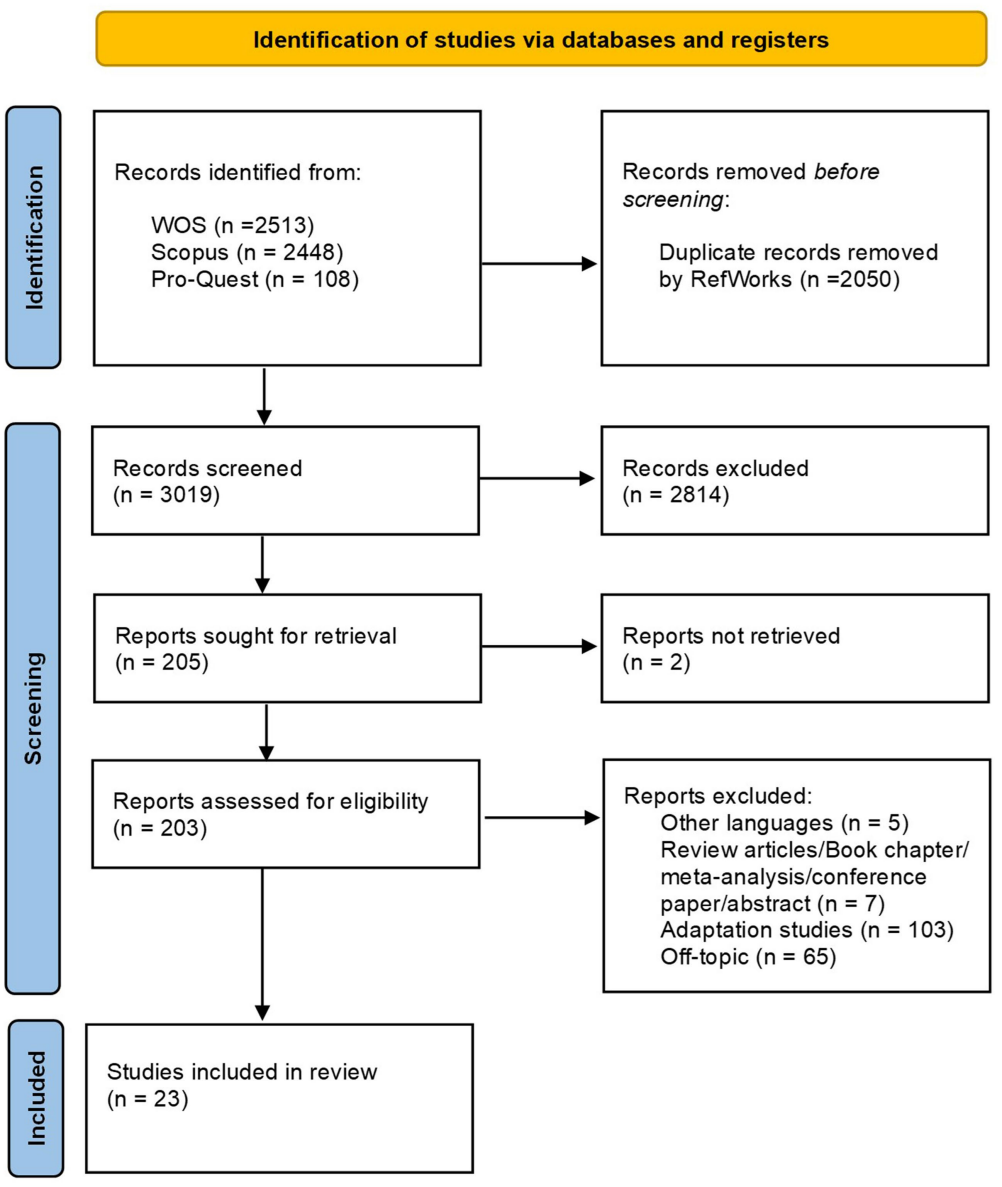
PRISMA flowchart. PRISMA flowchart showing the process used to identify search for meaning/quest for significance assessment tool studies.

Next, we sought to retrieve the full texts of the remaining 205 reports for assessment, and successfully obtained 203 of them, Of the 205 reports identified for full-text retrieval, two were unavailable due to restricted access or incomplete records, resulting in 203 reports being assessed. The full-text assessment led to the exclusion of 179 reports for reasons such as being in other languages (5 reports), being review articles, book chapters, meta-analyses, conference papers, or abstracts (7 reports), being adaptation studies (103 reports), and being off-topic (65 reports). Ultimately, 23 studies met all the inclusion criteria and were included in the systematic review. The Quest for Significance Scale (QFSS; Molinario et al., 2021) was not included in this study as it has not yet been officially published. The detailed study selection process is visually summarized in the PRISMA flow diagram shown in Figure 1.

### 2.4 Data mining

The following data were extracted from each included study, if available: (1) author (s)/year; (2) journal of publication; (3) scale name; (4) Scale language; (5) dimensions measured; (6) sample characteristics; and (7) psychometric properties.

### 2.5 Quality assessment

We employed the Strengthening the Reporting of Observational Studies in Epidemiology (STROBE) checklist (Vandenbroucke et al., 2007; Von Elm et al., 2007) and the Downs and Black checklist (Downs and Black, 1998) to comprehensively assess the quality of the included studies.

The Downs and Black checklist, comprising 27 items, assesses methodological quality across five dimensions: (1) general information, (2) external validity, (3) internal validity and biases, (4) internal validity and confounding variables, and (5) power. The STROBE checklist, consisting of 22 items, evaluates the reporting quality of observational studies. Both tools were scored independently by two reviewers, and inter-rater reliability was calculated using Cohen’s kappa (*κ* = 0.85), indicating strong agreement.

Studies were categorized based on their total scores: high quality (≥75% of the maximum score), moderate quality (50–74%), and low quality (<50%). This classification ensured a transparent and systematic evaluation of methodological rigor. Studies with higher quality scores were given greater weight in the synthesis of findings, as they were deemed more methodologically robust and reliable.

### 2.6 Bibliometric analysis

To conduct a comprehensive bibliometric analysis of the selected studies, we utilized VOSviewer software (Van Eck and Waltman, 2009). This analysis was conducted to identify key research trends, influential works, and collaboration patterns, which contribute to understanding gaps in measurement development and the evolution of research on the search for meaning and quest for significance.

The bibliometric analysis focused on three main types of networks: citation networks, co-authorship networks, and keyword co-occurrence networks.

Co-occurrence Keyword Network: We applied a minimum threshold of three keyword occurrences to identify major research themes.

Citation Network: A minimum threshold of 10 citations was set to identify influential works.

Co-citation Network: A minimum co-citation threshold of eight occurrences was applied to focus on significant relationships.

Co-authorship Network: A minimum threshold of two documents per author was used to highlight collaboration patterns. This bibliometric approach identified key themes, influential works, and collaboration patterns, providing a robust framework for understanding research on the search for meaning and quest for significance. These insights directly address the study’s primary research question by revealing gaps in existing measurement tools, such as underrepresented dimensions and insufficient cross-cultural validation. Hence, the bibliometric analysis is not merely a supplementary component of this study; it is a critical tool for identifying gaps in the literature and informing the development of more robust and contextually relevant assessment tools. By mapping the intellectual structure of the field, this analysis ensures that the systematic review is grounded in a comprehensive understanding of the research landscape, guiding future research toward addressing these gaps effectively.

## 3 Results

This systematic review incorporates 23 key studies that comprehensively evaluated instruments designed to measure the search for meaning in life and the quest for significance. The studies included populations spanning a variety of age ranges, from adolescents to adults, and were carried out in different cultural contexts, with instruments available in multiple languages. The tools that we assessed range from long-established measures to recently developed scales, ensuring a broad representation of approaches to assessing existential well-being. Each study was selected based on previously established inclusion criteria, and the analysis focused on their psychometric properties, including reliability, validity, and feasibility. Table 1 provides a detailed overview of all included studies and their specific characteristics, including the psychometric properties of the assessment tools that they assessed and the reliability, validity, and applicability of the tools in different populations. The strengths of each tool in terms of capturing the multidimensional aspects of meaning in life are discussed, as well as any limitations in their psychometric strength. Factors such as internal consistency, test–retest reliability, and construct validity were examined.

**Table 1:** Psychometric details of the included studies/tools.

Author(s)/year	Journal of publication	Scale name	Language	Dimensions measured	Sample characteristics	Psychometric properties
Balthip et al. (2022)	Kasetsart Journal of Social Sciences	Purpose in Life Scale for Thai Adolescents (PILTA)	English	Connectedness, meaning of life, self-worth, goal orientation, self-belief, determination, gratitude	*N* = 2,460, Thai students aged 15 to 19 years	Cronbach’s alpha of 0.92 + correlation with Seeking of Noetic Goals scale (r = 0.601) and the PIL scale (r = 0.597) (*p* = 0.01)—correlation with Beck hopelessness scale (r = −0.616) (*p* = 0.019)
Bellet et al. (2019)	Death Studies	Social Meaning in Life Events Scale (SMILES)	English	Social validation/invalidation	*N* = 590, college students	Cronbach’s alpha of 0.91 (Social Invalidation subscale), 0.84 (Social Validation subscale). Content Validity; Construct Validity: Two-factor structure Correlations with ISS, ISLES, and ICG-R
Campbell and Baumeister (1973)	The Journal of Psychology	Meaningfulness	English	Meaningfulness value	*N* = 24 children with intellectual disability *N* = 24 children without disability Average = 9.3	Test–Retest Reliability: Moderate; higher for children without disability and children with intellectual disability; correlations with adult values ranged from 0.60 to 0.75 Correlations of 0.77 (production method) 0.75 (paired-comparisons) for children without disability and children with intellectual disability
Caycho-Rodríguez et al. (2023)	Current Psychology	Purpose in Life Test—Short form (PIL-SF)	English	Purpose in life construct	*N* = 4,306 from seven Latin American countries, age range: 24.6 to 41.8	Reliability: Alpha coefficients ranging from 0.83 to 0.88; omega coefficients ranging from 0.84 to 0.87 Factor Analysis: Confirmatory Factor Analysis (CFA) using WLSMV estimator; model fit evaluated with chi-square, RMSEA, and SRMR
Chamberlain and Zika (1988)	Personality and Individual Differences	Purpose in Life (PIL) test	English	Purpose in life Sense of coherence Commitment and goal achievement Excitement life	*N* = 194 women having at least one child <5 years and no paid employment outside the home Average age = 29	NA
Chang and Dodder (1984)	The International Journal of Aging and Human Development	Modified Purpose in Life Test	English	Purpose in life Well-being	*N* = 177 retired American teachers Average age = 73 *N* = 202 retired Taiwanese teachers Average age = 67	Correlations: A positive and significant correlation with Affect Balance Scale (ABS): American sample: r = 0.30, *p* = 0.01 Taiwanese sample: r = 0.39, *p* = 0.01 Positive and significant correlations with positive affect schedule (PAS) and negative affect schedule (NAS)
Crumbaugh (1977)	Journal of Clinical Psychology	Seeking of Noetic Goals Test (SONG)	English	Strength of motivation to find meaning in life	Diverse groups; logotherapy patients, alcoholism treatment unit patients	NA
Fegg et al. (2008)	Journal of Pain and Symptom Management	The Schedule for Meaning in Life Evaluation (SMiLE)	English	Meaning in life Satisfaction	*N* = 599; university students	Convergent Validity: Significant correlations with: Purpose in Life Test (r = 0.48, *p* < 0.001) Self-Transcendence Scale (r = 0.34, *p* < 0.001) General numeric rating scale on Meaning in Life (r = 0.53, *p* < 0.001) Internal Consistency: Good; test–retest reliability of total weighted satisfaction (IoWS) at r = 0.72 (*p* < 0.001)
Fegg et al. (2016)	Palliative and Supportive Care	Schedule for Meaning in Life Evaluation (SMiLE)	English	Four major dimensions of meaning in life: leisure/health, work/finances, culture/spirituality, and relationships	*N* = 307; medical students Average age: 24.3	NA
George and Park (2017)	The Journal of Positive Psychology	Multidimensional Existential Meaning Scale (MEMS)	English	Comprehension Purpose Mattering	Sample 1: *N* = 188 Sample 2: *N* = 262 Sample 3: *N* = 160 Average age = 19	Reliability: Comprehension subscale: Cronbach’s alpha = 0.90 Mattering subscale: Cronbach’s alpha = 0.86
Hill et al. (2019)	Counselling Psychology Quarterly	Meaning in Life Measure (MILM)	English	Meaning in Life Measure (MILM) Two subscales: Experience (MILM-E) Reflectivity (MILM-R)	*N* = 401; American subjects	Internal Consistency: Cronbach’s alpha = 0.89 (MILM-E), 0.87 (MILM-R) Test–Retest Reliability: 0.82 (MILM-E), 0.79 (MILM-R)
Hutzell (1986)	The Hospice Journal	Life Purpose Questionnaire (LPQ)	English	Meaningfulness of life Purpose of life	*N* = 120	Test–Retest Reliability: High in the original study (r = 0.90); assessments with other populations are lacking
Li et al. (2021)	Journal of Happiness Studies	Quadripartite Existential Meaning Scale (QEMS)	English	Meaning in life (MIL): Feelings of coherence, purpose, and external value (significance or mattering) and internal value	Sample 1: *N* = 201, mean age = 19.9 Sample 2: *N* = 336, mean age = 20.3	Internal Consistency: Comprehension: *ω* = 0.88, *α* = 0.87 Purpose: ω = 0.91, α = 0.91 Internal Value (IV): ω = 0.91, α = 0.91 External Value (EV): ω = 0.89, α = 0.88 Test–Retest Reliability: Correlations after 4 weeks: Comprehensio*N* = 0.65, Purpose = 0.74, IV = 0.72, EV = 0.74
Martela and Steger (2023)	The Journal of Positive Psychology	Three-Dimensional Meaning in Life Scale (3DM)	English	Meaning in Life: Significance Purpose Coherence	Study 1: *N* = 301; Study 2: *N* = 300; Study 3: *N* = 171; Study 4: *N* = 241; Study 5: *N* = 336 Age range = 19 to 71	Internal Consistency: Coherence: Cronbach’s alpha = 0.89 Purpose: Cronbach’s alpha = 0.91 Significance: Cronbach’s alpha = 0.89
Molina et al. (2023)	CES Psicología	Life Purpose Scale for Adolescents	Spanish	Search for Purpose in Life Identification of Purpose in Life	*N* = 554; mean age = 15.32 Argentina	Reliability: Search Component: Cronbach’s alpha = 0.85 Identification Component: Cronbach’s alpha = 0.91 Overall: Cronbach’s alphas ranging from 0.87 to 0.92 Search Component: Explained 74.9% of the variance; Goodness of Fit Index (GFI) = 1.00
Reker and Peacock (1981)	Canadian Journal of Behavioural Science	Life Attitude Profile (LAP)	English	Life Purpose, Existential Vacuum, Life Control, Death Acceptance, Will to Meaning, Goal Seeking, Future Meaning to Fulfil	*N* = 219 students; mean age = 21.6	Higher-Order Factors: Account for 48 and 67% of total variance Internal Consistency: Ranges from 0.83 (Life Purpose) to 0.55 (Future Meaning to Fulfil) Factor-to-Composite Correlations: Ranges from 0.34 (Existential Vacuum) to 0.62 (Goal Seeking), all significant at *p* < 0.001
Şahi̇n and Deri̇n (2023)	International Journal of Educational Research Review	Significance Quest Scale (SQS)	English	Quest for significance in Life	*N* = 621; age > 18 Turkish subjects	Internal Consistency: Cronbach’s alpha = 0.95 Convergent Validity: 0.67
Salsman et al. (2020)	Quality of Life Research	Meaning and Purpose in Life (PROMIS)	English	Meaning and Purpose, Life satisfaction	*N* = 1,000; mean age = 47.8 Included a wide range of age groups	Internal Consistency: 4-item Short Form: α = 0.90 37-item Bank: α = 0.98
Scheier et al. (2006)	Journal of Behavioral Medicine	Life Engagement Test (LET)	English	Purpose in life	*N* = 2,076 total in eight samples consisting of adults, students, and patients	Test–Retest Stability: Moderate, with correlations ranging from 0.61 to 0.76 Convergent Validity: Correlates with various psychosocial measures and health-relevant variables
Schnell (2009)	Journal of Positive Psychology	Sources of Meaning and Meaning in Life questionnaire (SoMe)	English	Meaningfulness (5 items), Crisis of Meaning (5 items), and 26 sources of meaning grouped into four higher-order dimensions: Self-transcendence (vertical/horizontal), Self-actualization, Order, and Well-being/Relatedness	*N* = 616; range of age (16–85); Mean age = 45 German subjects	Internal consistency: α = 0.83–.93 for the four higher-order dimensions; *α* = 0.65–0.95 for the 26 source scales (German representative sample, *N* = 603). Two-month test–retest coefficients average .81 for the scales and 0.90 for the dimensions; six-month stability 0.72/0.78 for sources and dimensions (crisis of meaning = 0.48). Extensive evidence of construct, content, discriminant, factorial, and incremental validity has been reported
Schnell and Danbolt (2023)	BMC Psychology	Meaning and Purpose Scales (MAPS)	English	Meaningfulness, Crisis of Meaning, and five “sources of purpose” scales: Sustainability, Faith, Security, Community, and Personal Growth	*N* = 974; range of age (18–89); mean age = 50 German subjects	Population-based German sample (N = 974); *α* = 0.74–0.96, *ω* = 0.75–0.96; all corrected item–total rs > 0.52; EFA/CFA support 2-factor (Meaningfulness, Crisis) and 5-factor (purpose) structure; test–retest ≥ 0.75 at 4 weeks and 2 months; convergent/divergent validity with SoMe, criterion validity (Sustainability with pro-environmental behaviour), and predictive validity for general mental distress
Steger et al. (2006)	Journal of Counseling Psychology	Meaning in Life Questionnaire (MLQ)	English	Presence of Meaning Search for Meaning	*N* = 70; mean age = 20.1 Multiple ethnicities	Internal Consistency: Presence of Meaning: α = 0.86 Search for Meaning: α = 0.88 for both Positive Correlations: Life satisfaction, positive emotions, intrinsic religiosity, extraversion, agreeableness Negative Correlations: Depression, negative emotions, neuroticism
Zambelli and Tagliabue (2024)	Journal of Happiness Studies	Situational Meaning in Life Evaluation (SMILE)	English	Comprehension Significance Purpose Presence and Search for meaning	Study 1 & Study 2: *N* = 3,035 Mean age = 48.3 Study 3: *N* = 283; Mean age = 26 Italian subjects	Reliability: Presence of Meaning: Ω = 0.84 Search for Meaning: Ω = 0.83 Convergent Validity: CFI = 0.93

### 3.1 Dimensions measured

The studies included in this systematic review focus on a variety of dimensions related to the search for meaning in life and the quest for significance. Key dimensions measured across the different assessment tools include existential well-being, purpose in life, personal significance, social relevance, and cosmic significance. For instance, the Purpose in Life (PIL) test (Chamberlain and Zika, 1988) and its short form (PIL-SF) (Caycho-Rodríguez et al., 2023) evaluate the extent to which individuals perceive their lives as being purposeful and meaningful. The Social Meaning in Life Events Scale (Bellet et al., 2019) explores how social interactions contribute to one’s sense of meaning, while the Multidimensional Existential Meaning Scale (MEMS) (George and Park, 2017) assesses personal, social, and cosmic dimensions of existential meaning. The Significance Quest Scale (SQS) (Şahi̇n and Deri̇n, 2023), on the other hand, specifically assesses an individual’s drive for significance in personal, social, and cosmic contexts, aligning closely with Kruglanski’s theory of the quest for significance, which posits that individuals are motivated to achieve a sense of importance and value in their lives. These dimensions are crucial for understanding how different aspects of life contribute to an individual’s overall sense of meaning and significance.

However, a critical evaluation of these tools reveals gaps in their ability to fully capture the dynamic and context-dependent nature of the quest for significance. For example, while the SQS aligns well with Kruglanski’s theory, it may not adequately address the role of cultural and situational factors in shaping an individual’s drive for significance. Similarly, the MEMS, though comprehensive, lacks specificity in measuring the intensity of the quest for significance in different life domains.

### 3.2 Sample characteristics

The samples of the included studies were diverse, encompassing a wide range of populations in terms of age, cultural background, and life stage. The studies involve participants from adolescence to older adulthood, reflecting the universal relevance of the search for meaning and quest for significance. For example, the Purpose in Life Scale for Thai Adolescents (Balthip et al., 2022) was designed for use with young people in Thailand, addressing cultural specificities and developmental stages unique to adolescents. In contrast, the Life Purpose Scale for Adolescents by Molina et al. (2023) is administered to Spanish-speaking adolescents, highlighting the tool’s adaptability across languages and cultures. Other tools, such as the Meaning in Life Questionnaire (MLQ) published by Steger et al. (2006), are widely used across different demographic groups, demonstrating broad applicability.

The Meaning and Purpose Scales (MAPS; Schnell & Danbolt, 2023) were validated in several large German samples, including a development study with *N* = 13,660 participants and a population-based sample of adults (*N* = 974). The authors report good to excellent internal consistency for all scales (Cronbach’s α = 0.74–0.96; McDonald’s ω = 0.75–0.96), corrected item–total correlations above 0.52, and stable two- and five-factor structures for meaning and sources-of-purpose scales confirmed by CFA across gender and age groups. Test–retest reliability over 4 weeks and 2 months was at least 0.75 for every scale, and extensive evidence of convergent and divergent validity with the SoMe, criterion validity (e.g., Sustainability predicting pro-environmental behaviour), and predictive validity for general mental distress was provided.

This diversity ensures that the findings from these studies can be generalized to various populations, enhancing the overall robustness and inclusivity of our systematic review. However, it also highlights the need for further research into how cultural and demographic differences influence the validity and effectiveness of these tools. For instance, while the MLQ has been validated in multiple cultural contexts, its applicability to non-Western populations remains limited due to its emphasis on individualistic notions of meaning and purpose.

#### 3.2.1 Psychometric Properties.

The psychometric properties of the assessment tools included in this review were rigorously evaluated to ensure their reliability and validity. Key properties assessed include internal consistency, test–retest reliability, construct validity, and criterion validity. The Downs and Black checklist and the STROBE checklist were employed to assess the methodological quality of the studies, ensuring a comprehensive evaluation of their psychometric soundness. For instance, the PIL (Chamberlain and Zika, 1988) and PIL-SF tests (Caycho-Rodríguez et al., 2023) demonstrate high internal consistency and good test–retest reliability, making them reliable tools for longitudinal studies. The SQS (Şahi̇n and Deri̇n, 2023) shows strong construct validity, aligning well with Kruglanski’s theoretical framework. Moreover, tools like the MEMS (George and Park, 2017) and the MLQ (Steger et al., 2006) have been validated in different cultural contexts, demonstrating their criterion validity. However, a side-by-side comparison of these tools reveals notable strengths and weaknesses. For example, while the PIL and PIL-SF are robust in measuring purpose in life, they may not fully capture the multidimensional nature of existential meaning, as addressed by the MEMS. Similarly, the MLQ, though widely used, has been critiqued for its limited ability to measure the intensity of the quest for significance, a gap that the SQS attempts to address. Quality Assessment.

To appraise the methodological quality of the included studies, we used two complementary checklists. First, the Downs and Black Quality Index was applied to the 20 empirical studies that reported quantitative analyses of associations or group differences, regardless of whether their design was strictly experimental or non-experimental. This instrument was originally developed to assess the methodological quality of randomized and non-randomized intervention studies, but most of its items refer to generic aspects of study quality (e.g., clarity of hypotheses and outcomes, description of participants, use of appropriate statistics). In non-experimental and psychometric validation studies, items that referred specifically to random allocation, blinding or follow-up were difficult to apply and were scored conservatively as 0, which likely leads to an underestimation of certain aspects of methodological rigour in these designs. Second, for the three studies that were purely observational and descriptive (Chamberlain and Zika, 1988; Crumbaugh, 1977; Fegg et al., 2016), we did not use the Downs and Black Index and instead assessed reporting quality using the STROBE checklist. In our synthesis, higher Downs and Black scores were interpreted as indicating better overall methodological quality among the analytical studies, whereas higher STROBE adherence reflected more comprehensive and transparent reporting in the descriptive observational studies.

For the three non-experimental studies, namely Chamberlain and Zika (1988), Crumbaugh (1977), and Fegg et al. (2016), we utilized the STROBE checklist and found that the total scores ranged from 16 to 19.

The detailed outcomes of the quality assessments for the experimental studies, conducted using the Downs and Black checklist, are summarized in Table 2. This table provides a clear and structured evaluation of the methodological quality of these studies. This dual assessment approach ensures a rigorous and systematic evaluation of the included studies, thereby enhancing the credibility and reliability of our systematic review findings.

**Table 2:** Summary of study quality assessments performed using the Downs and Black and STROBE checklists.

Study	Quality Assessment Tool					
	Downs and Black checklist					
	Overall quality max score = 11	External validity max score = 3	Internal validity bias max score = 7	Internal validity confusion max score = 6	Power max score = 5	Score out of 32
Balthip et al. (2022)	7	3	5	6	5	26
Bellet et al. (2019)	10	2	3	1	5	21
Campbell and Baumeister (1973)	7	2	3	1	2	15
Caycho-Rodríguez et al. (2023)	9	2	4	3	5	23
Chang and Dodder (1984)	5	2	3	0	3	13
Fegg et al. (2008)	7	2	4	2	4	19
George and Park (2017)	9	3	5	6	5	28
Hill et al. (2019)	6	3	2	0	4	15
Hutzell (1986)	7	0	2	0	3	12
Li et al. (2021)	7	2	2	2	3	16
Martela and Steger (2023)	8	2	2	1	5	18
Molina et al. (2023)	7	3	3	2	2	17
Reker and Peacock (1981)	9	3	6	4	4	26
Şahi̇n and Deri̇n (2023)	8	0	3	0	4	15
Salsman et al. (2020)	7	3	3	0	4	17
Scheier et al. (2006)	9	2	2	1	4	18
Schnell (2009)	9	2	3	1	5	20
Schnell and Danbolt (2023)	8	3	3	0	5	19
Steger et al. (2006)	9	2	3	1	5	20
Zambelli and Tagliabue (2024)	7	3	3	0	4	17

	STROBE checklist						
Study	Title and summary max score = 1	Introduction max score = 2	Methods max score = 9	Results max score = 5	Discussion max score = 4	Other information max score = 1	Score out of 22
Chamberlain and Zika (1988)	1	2	8	4	4	0	19
Crumbaugh (1977)	1	2	7	5	3	0	18
Fegg et al. (2016)	1	2	7	3	3	0	16

### 3.3 Bibliometric analysis

The bibliometric analysis provides a comprehensive overview of the intellectual structure and research trends in the field of assessment tools for the search for meaning and the quest for significance. By analyzing co-occurrence keyword networks, citation networks, co-citation networks, and co-authorship networks, we identified key themes, influential works, and collaboration patterns. These insights not only map the evolution of the field but also highlight gaps in the literature and inform the development of more robust assessment tools. Importantly, this analysis directly contributes to the study’s central question by identifying areas where existing tools may be insufficient and guiding future research toward addressing these gaps.

#### 3.3.1 Co-occurrence keyword network

**Figure 2:**
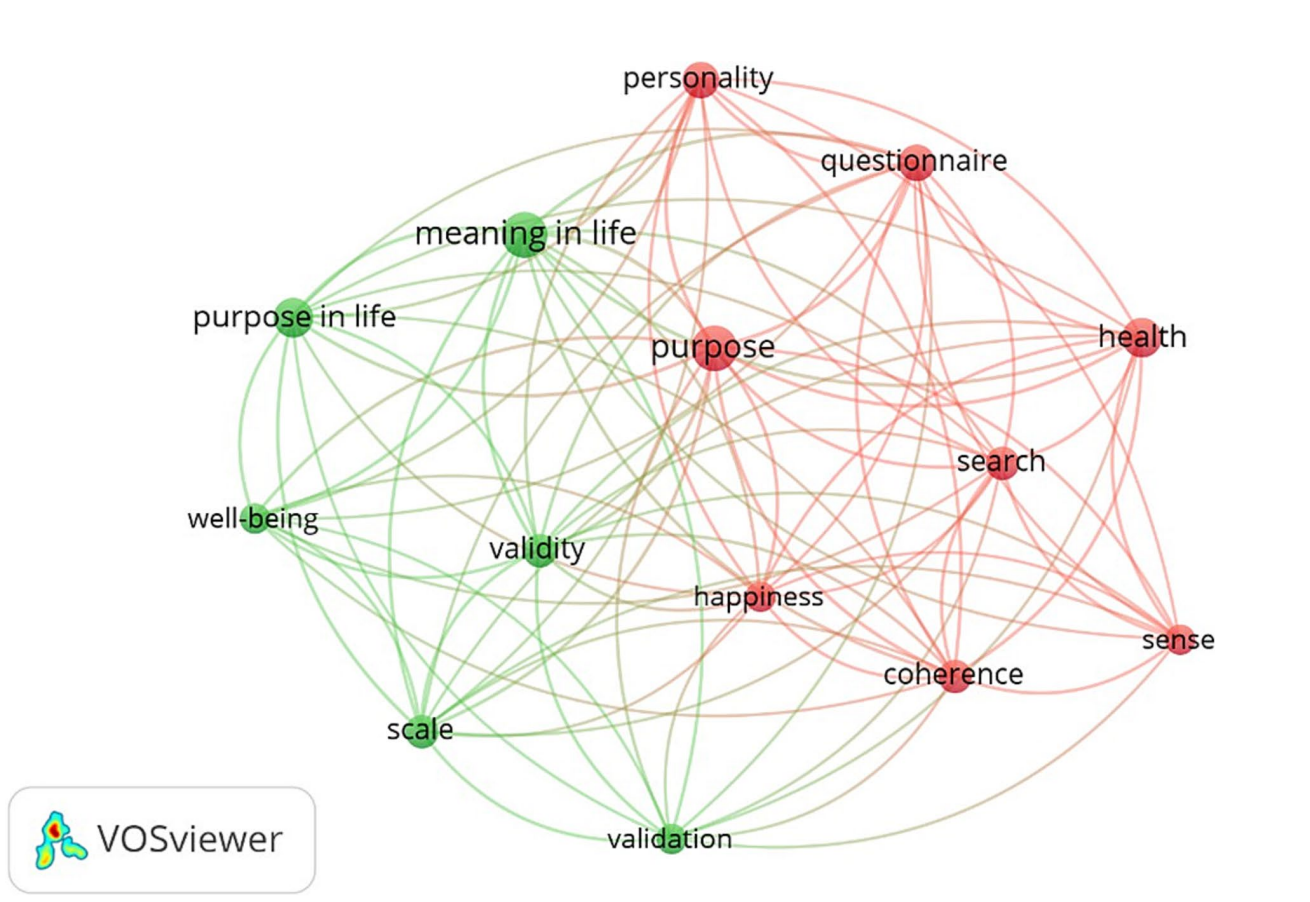
Co-occurrence keyword network map.

The co-occurrence keyword network map illustrates the connections between the most frequently occurring keywords in studies of search for meaning assessment tools (Figure 2). Notable nodes such as “purpose,” “meaning in life,” and “health” are central to the network, indicating their high degree of relevance and frequent discussion within the literature. The keyword “purpose” serves as a pivotal node that connects both clusters, highlighting its crucial role in the discourse on assessment tools for the search for meaning. The keyword “meaning in life” is particularly significant within the green cluster, emphasizing its importance in the context of well-being and life purpose research. The network reveals a strong interconnection between psychological well-being and the search for life’s purpose, suggesting a multidisciplinary approach to understanding how purpose and meaning in life contribute to health and psychological outcomes. Additionally, the presence of validation-related keywords indicates ongoing efforts to develop and refine measurement tools in this research area, which is integral to the study of assessment tools for the search for meaning.

The network consists of two distinct clusters, each representing a different thematic area. The red cluster includes keywords such as “personality,” “questionnaire,” “health,” “search,” “sense,” “coherence,” and “purpose.” This cluster represents research themes related to the psychological and health aspects of the search for meaning, focusing on personality assessments, health implications, and the search for purpose and meaning. In contrast, the green cluster comprises keywords such as “purpose in life,” “meaning in life,” “well-being,” “validity,” “scale,” and “validation.” This cluster is centered on research themes involving the well-being and validation measures associated with finding purpose and meaning in life.

#### 3.3.2 Citation network

**Figure 3:**
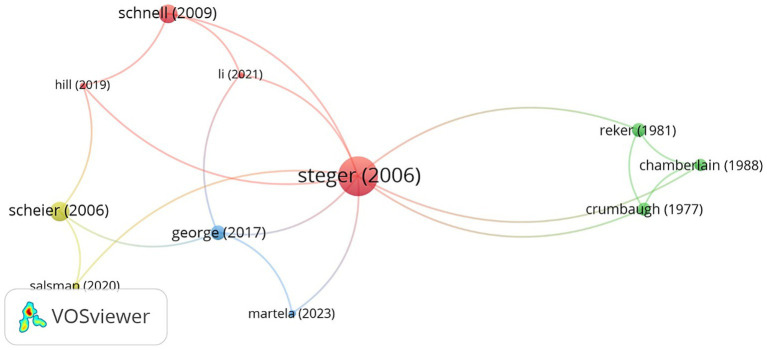
Citation network map. The size of the nodes represents the frequency of citations, and the thickness of the links indicates the strength of the citation relationships.

The citation network map (Figure 3) illustrates the connections between highly cited works exploring search for meaning assessment tools. The network highlights several key publications that serve as pivotal nodes, indicating their significant influence and frequent citation within the literature. Notably, “Steger et al. (2006)” emerges as the most central and frequently cited work, reflecting its foundational role in this research domain.

The citation network reveals a strong interconnection between foundational works and contemporary studies on assessment tools for the search for meaning. This indicates a multidisciplinary approach, combining psychological assessment, health implications, and the search for meaning. The network underscores the evolution of the field, from early foundational studies to modern advancements, highlighting the significant contributions of key publications to the discourse on assessment tools and psychometric aspects related to the search for meaning.

The network consists of several clusters, each representing different research traditions and influential works in the field. The red cluster, centered around “Steger et al. (2006),” includes significant studies such as “Schnell (2009),” “Hill et al. (2019),” and “Li et al. (2021).” These works primarily focus on the development and validation of meaning and purpose assessment tools, as well as their psychological implications. The green cluster comprises earlier foundational research like “Reker and Peacock (1981),” “Chamberlain and Zika (1988),” and “Crumbaugh (1977),” which focus on the conceptualization and measurement of meaning in life. Additionally, the yellow cluster includes works like “Scheier et al. (2006)” and “Salsman et al. (2020),” which explore psychological assessments and their applications in health and well-being, while the blue cluster features recent advancements, with studies like “George and Park (2017)” and “Martela and Steger (2023).”

#### 3.3.3 Co-citation network

**Figure 4:**
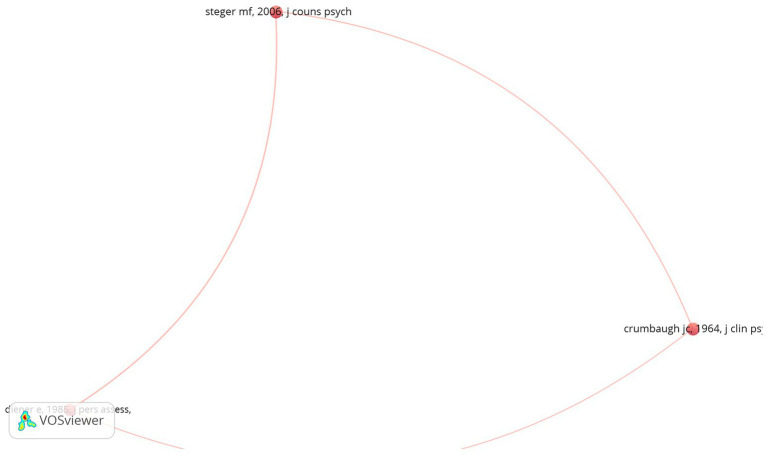
Co-citation network map. The size of the nodes represents the frequency of co-citations, and the thickness of the links indicates the strength of these relationships.

The co-citation network map (Figure 4) illustrates the relationships between frequently co-cited papers on assessment tools for the search for meaning. The map highlights significant works that are often cited together, indicating their combined influence on the field. Notably, “Steger et al. (2006)” and “Crumbaugh and Maholick (1964)” emerge as the most central and frequently co-cited works, signifying their foundational roles and interconnected influence in this research domain. Additionally, “Diener et al. (1985)” is another highly co-cited work, reflecting its importance in the context of well-being and life satisfaction research. The network reveals key clusters that bridge various research traditions, with “Steger et al. (2006)” and “Crumbaugh and Maholick (1964)” central to the development and validation of meaning and purpose assessment tools, while “Diener et al. (1985)” was foundational for well-being research. The strong co-citation links underscore the interdisciplinary nature of research in this field, combining psychological assessment, health implications, and the search for meaning, highlighting the ongoing relevance of these seminal works in advancing the field’s methodological rigor.

Co-citation network map of frequently co-cited papers on assessment tools for the search for meaning. The size of the nodes represents the frequency of co-citations, and the thickness of the links indicates the strength of these relationships. Notably, “Steger et al. (2006),” “Crumbaugh and Maholick (1964),” and “Diener et al. (1985)” are the most central and frequently co-cited works, highlighting their foundational roles and interconnected influence in this research domain.

#### 3.3.4 Co-authorship network

**Figure 5:**
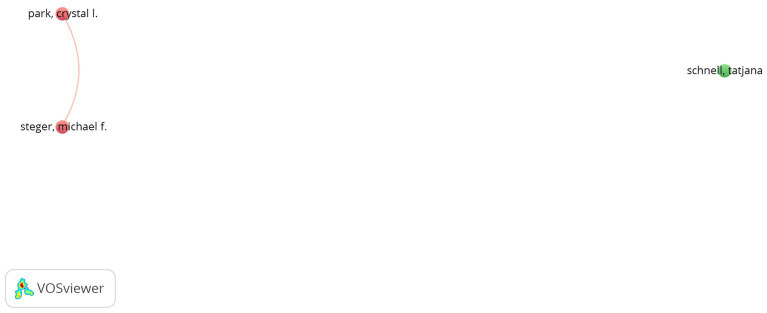
Co-authorship network map. The size of the nodes represents the number of publications by each author, and the thickness of the links indicates the strength of their co-authorship ties.

The co-authorship network map (Figure 5) illustrates the collaborative relationships among researchers in the field of assessment tools for the search for meaning. The map highlights three key authors: Michael F. Steger, Crystal L. Park, and Tatjana Schnell. Steger and Park show a direct co-authorship link, indicating a collaborative relationship, while Schnell is more isolated, reflecting a less connected but still significant contribution. This network provides insights into the collaboration patterns and influential researchers within the field.

### 3.4 Practical applications of assessment tools

The assessment tools reviewed in this study have demonstrated significant utility in both clinical practice and research contexts, offering valuable insights into the search for meaning and existential well-being. For example, the Meaning in Life Questionnaire (MLQ) (Steger et al., 2006) is widely used in clinical settings to assess the presence of and search for meaning, providing a foundation for therapeutic interventions aimed at enhancing existential well-being. Its strong psychometric properties, including high internal consistency (α = 0.86 for Presence of Meaning and α = 0.88 for Search for Meaning), make it a reliable tool for both individual and group therapy. Similarly, the Multidimensional Existential Meaning Scale (MEMS) (George and Park, 2017) is highly effective in research settings, offering a comprehensive assessment of comprehension, purpose, and mattering, which are critical for understanding the multifaceted nature of existential meaning.

In clinical practice, tools like the Purpose in Life (PIL) test (Chamberlain and Zika, 1988) and its short form (PIL-SF) (Caycho-Rodríguez et al., 2023) are particularly useful for evaluating life purpose and coherence, guiding interventions for individuals experiencing existential distress. These tools have been validated across diverse populations, including adolescents and older adults, making them adaptable to various clinical contexts. Additionally, the Social Meaning in Life Events Scale (SMILES) (Bellet et al., 2019) provides valuable insights into the role of social interactions in shaping meaning, which can inform social support interventions in clinical settings.

In research, tools like the Significance Quest Scale (SQS) (Şahi̇n and Deri̇n, 2023) and the Three-Dimensional Meaning in Life Scale (3DM) (2023) are well-suited for exploring the intensity of the quest for significance and the interplay between coherence, purpose, and significance. These tools have shown strong construct validity and reliability, making them valuable for studies on existential motivation and well-being. However, while these instruments are highly effective for measuring the search for meaning, their ability to fully capture the dynamic and context-dependent nature of the quest for significance remains limited. For example, the SQS, though aligned with Kruglanski’s theory, may not adequately address cultural and situational factors that influence the drive for significance. Similarly, the MLQ, while robust in assessing existential well-being, does not explicitly measure the intensity of the quest for significance, highlighting a gap in the current measurement landscape.

Overall, the reviewed tools offer substantial benefits for clinical practice and research, providing reliable and valid measures of existential well-being and life purpose. However, their limitations in capturing the quest for significance suggest a need for further development of tools that integrate psychological and existential dimensions of meaning, ensuring their applicability across diverse contexts.

## 4 Discussion

This systematic review highlights the strengths and limitations of existing assessment tools designed to measure the search for meaning in life and the quest for significance. While these instruments adeptly capture various dimensions of existential well-being, they fall short in precisely measuring the quest for significance as conceptualized by the theory of Kruglanski et al.’s (2022). Our findings reveal that existing tools, such as the MLQ (Steger et al., 2006) and the PIL test (Chamberlain and Zika, 1988), primarily focus on the presence of and search for meaning but lack specificity in assessing the nuanced facets of the quest for significance, particularly social relevance and cosmic significance. This gap underscores the need for a more targeted and comprehensive measurement tool that aligns closely with Kruglanski’s theoretical framework.

### 4.1 Critique of existing tools

A detailed critique of existing tools reveals several limitations. For example, the MLQ, while widely used and psychometrically robust, does not adequately capture the intensity of the quest for significance in social and cosmic contexts. Similarly, the PIL test, though effective in assessing purpose in life, fails to account for the dynamic and context-dependent nature of the quest for significance. These tools often conflate the search for meaning with the quest for significance, limiting their ability to differentiate between these distinct constructs. For instance, the Significance Quest Scale (SQS) (Şahi̇n and Deri̇n, 2023) demonstrates good reliability and initial validity but is limited by its narrow sample (Turkish population, mean age = 34.41 years), raising concerns about its cross-cultural applicability and generalizability across life stages.

Furthermore, existing tools lack sensitivity to situational fluctuations in the quest for significance, a critical feature for understanding how immediate experiences, such as loss or gain of significance, influence behavior. For example, while the Multidimensional Existential Meaning Scale (MEMS) (George and Park, 2017) comprehensively assesses comprehension, purpose, and mattering, it does not explicitly measure the intensity of the quest for significance or its situational variability. These limitations highlight the need for a new tool that integrates psychological and existential dimensions of meaning while remaining sensitive to cultural and contextual factors.

### 4.2 Cross-cultural validation

Cross-cultural validation is a critical area where existing tools fall short. While instruments like the MLQ and MEMS have been validated in multiple cultural contexts, their applicability to non-Western populations remains limited due to their emphasis on individualistic notions of meaning and purpose. For example, the Purpose in Life Scale for Thai Adolescents (PILTA) (Balthip et al., 2022) addresses cultural specificities unique to Thai adolescents but has not been widely tested in other non-Western contexts. Similarly, the Life Purpose Scale for Adolescents (Molina et al., 2023) demonstrates adaptability across languages but lacks validation in diverse cultural settings.

These gaps in cross-cultural validation suggest that existing tools may not fully capture the quest for significance in non-Western populations, where cultural and social factors may shape the experience of significance differently. For instance, in collectivist cultures, social relevance may play a more central role in the quest for significance than in individualistic cultures. Future tools must be rigorously tested across diverse cultural contexts to ensure their universal applicability and cultural sensitivity.

### 4.3 Age and life stage considerations

The age distribution of study samples also reveals important limitations. While tools like the PILTA and the Life Purpose Scale for Adolescents are designed for specific age groups, most existing instruments, such as the MLQ and MEMS, are validated primarily in adult populations. This raises concerns about their applicability across different life stages, particularly adolescence and older adulthood, where the experience of meaning and significance may differ significantly.

For example, adolescents may conceptualize the quest for significance in terms of identity formation and social validation, while older adults may focus on legacy and existential fulfillment. A new measurement tool must account for these variations by incorporating age-specific dimensions and ensuring sensitivity to the unique challenges and motivations associated with different life stages.

### 4.4 Integration of bibliometric findings

The bibliometric analysis provides valuable insights into the intellectual structure of the field and highlights gaps in the literature. Key works such as Steger et al. (2006) and Crumbaugh and Maholick (1964) dominate the citation network, reflecting their foundational role in the development of meaning in life assessment tools. However, the lack of diversity in influential studies, particularly from non-Western and culturally diverse populations, underscores the need for greater exploration of underrepresented perspectives.

The bibliometric findings also reveal a lack of integration between research on the search for meaning and the quest for significance. For example, while Steger et al. (2006) and Diener et al. (1985) are frequently co-cited, their focus on well-being and life satisfaction does not fully address the motivational drive for significance as conceptualized by Kruglanski. This gap in the literature supports the argument for a new scale that bridges these research traditions and provides a more comprehensive assessment of the quest for significance.

### 4.5 Key requirements for a new measurement tool

Based on our findings, we propose the following key requirements for an ideal measurement tool:

Multidimensional Assessment: The tool should capture the three facets of the quest for significance—personal significance, social relevance, and cosmic significance—as outlined by Kruglanski’s theory.

Cross-Cultural Validity: The tool must be rigorously validated across diverse cultural contexts to ensure universal applicability and cultural sensitivity.

Sensitivity to Situational Contexts: The tool should be able to capture fluctuations in the quest for significance based on situational factors, such as loss or gain of significance.

Age-Specific Adaptability: The tool should account for variations in the experience of significance across different life stages, from adolescence to older adulthood.

Strong Psychometric Properties: The tool must demonstrate high internal consistency, test–retest reliability, discriminant validity, and predictive validity to ensure its reliability and accuracy.

By incorporating these features, the new tool would significantly enhance our understanding of the quest for significance and its implications for behavior, well-being, and interventions.

### 4.6 Future research directions

To address the limitations of existing tools, we recommend the following specific steps for future research:

Refine the SQS: Conduct factor analysis and expand validation studies to include diverse cultural and age groups.

Develop a New Scale: Pilot a new scale that integrates psychological and existential dimensions of meaning, with a focus on sensitivity to situational and cultural contexts.

Test Situational Sensitivity: Use experimental manipulations to test the tool’s ability to capture fluctuations in the quest for significance.

Conduct Cross-Cultural Studies: Validate the new tool in diverse cultural settings to ensure its universal applicability.

Explore Age-Specific Variations: Investigate how the quest for significance is conceptualized and experienced across different life stages.

These steps will fill critical gaps in the literature and provide a robust foundation for future research on the quest for significance.

## 5 Conclusion

This systematic review evaluated existing tools for measuring the search for meaning and the quest for significance, revealing significant gaps in capturing the latter as a distinct construct. While instruments like the MLQ (Steger et al., 2006) and PIL test (Chamberlain and Zika, 1988) effectively assess existential well-being, they lack specificity in measuring the quest for significance—particularly its dimensions of personal significance (intrinsic self-worth), social relevance (validation from others), and cosmic significance (broader existential or spiritual meaning). This limitation conflates the two constructs, reducing measurement accuracy and obscuring their unique effects on behavior and well-being. The bibliometric analysis further highlights this gap, showing that foundational works like Steger et al. (2006) dominate the field but fail to integrate research on the quest for significance, underscoring the need for a new scale. We propose a dedicated Quest for Significance Scale that explicitly measures personal, social, and cosmic significance; is rigorously validated across diverse cultural contexts to ensure universal applicability; captures situational fluctuations in the quest for significance (e.g., loss or gain of significance); and adapts to different life stages, addressing how adolescents and older adults may conceptualize significance differently. Such a tool would not only fill critical gaps in the literature but also enhance research and practice by providing a robust foundation for understanding the quest for significance and its implications for psychological well-being, resilience, and behavior.

## Data Availability

The original contributions presented in the study are included in the article/supplementary material, further inquiries can be directed to the corresponding author.
