# Correction: Systematic review of search for meaning in life assessment tools: highlighting the need for a quest for significance scale

**DOI:** 10.3389/fpsyg.2026.1809072

**Published:** 2026-04-08

**Authors:** Fahd Alsaadi, Miguel A. Maldonado, Mohammad Erfanikia, Erica Molinario, Manuel Moyano

**Affiliations:** 1 Departamento de Psicología, Facultad de Ciencias de la Educación y Psicología, Universidad de Córdoba, Córdoba, Spain; 2 Hospital Universitario “Reina Sofía”, Instituto Maimónides de Investigación Biomédica de Córdoba, Córdoba, Spain; 3 School of Psychology, University of East Anglia, Norwich, United Kingdom; 4 Department of Psychology, Florida Gulf Coast University, Fort Myers, FL, United States; 5 Institute of Psychology, Jagiellonian University, Kraków, Poland

**Keywords:** quest for significance, meaning in life, search for meaning, psychometric assessment, bibliometric analysis, systematic review

There was a mistake in Table 1 as published.

**Table 1:** Psychometric details of the included studies/tools.

Author(s)/ year	Journal of publication	Scale name	Language	Dimensions measured	Sample characteristics	Psychometric properties
Balthip et al. (2022)	Kasetsart Journal of Social Sciences	Purpose in Life Scale for Thai Adolescents (PILTA)	English	Connectedness, meaning of life, self-worth, goal orientation, self-belief, determination, gratitude	*N* = 2,460, Thai students aged 15–19 years	Cronbach's alpha of 0.92 + correlation with Seeking of Noetic Goals scale (*r* = 0.601) and the PIL scale (*r* = 0.597) (*p* = 0.01—correlation with Beck hopelessness scale (*r* = −0.616) (*p* = 0.019)
Bellet et al. (2019)	Death Studies	Social Meaning in Life Events Scale (SMILES)	English	Social validation/ invalidation	*N* = 590, college students	Cronbach's alpha of 0.91 (Social Invalidation subscale), 0.84 (Social Validation subscale). Content Validity; Construct Validity: Two-factor structure Correlations with ISS, ISLES, and ICG-R
Campbell and Baumeister (1973)	The Journal of Psychology	Meaningfulness	English	Meaningfulness value	*N* = 24 children with intellectual disability *N* = 24 children without disability Average = 9.3	Test-retest reliability: Moderate; higher for children without disability and children with intellectual disability; correlations with adult values ranged from 0.60 to 0.75 Correlations of 0.77 (production method) 0.75 (paired-comparisons) for children without disability and children with intellectual disability
Caycho-Rodríguez et al. (2023)	Current Psychology	Purpose in Life Test-Short form (PIL-SF)	English	Purpose in life construct	*N* = 4,306 from seven Latin American countries, age range: 24.6–41.8	Reliability: Alpha coefficients ranging from 0.83 to 0.88; omega coefficients ranging from 0.84 to 0.87 Factor analysis: Confirmatory Factor Analysis (CFA) using WLSMV estimator; model fit evaluated with chi-square, RMSEA, and SRMR
Chamberlain and Zika (1988)	Personality and Individual Differences	Purpose in Life (PIL) test	English	Purpose in life Sense of coherence Commitment and goal achievement Excitement life	*N* = 194 women having at least one child < 5 years and no paid employment outside the home Average age = 29	NA
Chang and Dodder (1984)	The International Journal of Aging and Human Development	Modified Purpose in Life Test	English	Purpose in life Well-being	*N* = 177 retired American teachers Average age = 73 *N* = 202 retired Taiwanese teachers Average age = 67	Correlations: A positive and significant correlation with Affect Balance Scale (ABS): American sample: *r* = 0.30, *p* = 0.01 Taiwanese sample: *r* = 0.39, *p* = 0.01 Positive and significant correlations with positive affect schedule (PAS) and negative affect schedule (NAS)
Crumbaugh (1977)	Journal of Clinical Psychology	Seeking of Noetic Goals Test (SONG)	English	Strength of motivation to find meaning in life	Diverse groups; logotherapy patients, alcoholism treatment unit patients	NA
Fegg et al. (2008)	Journal of Pain and Symptom Management	The Schedule for Meaning in Life Evaluation (SMiLE)	English	Meaning in life Satisfaction	*N* = 599; university students	Convergent validity: Significant correlations with: Purpose in Life Test (*r* = 0.48, *p* < 0.001) Self-Transcendence Scale (*r* = 0.34, *p* < 0.001) General numeric rating scale on Meaning in Life (*r* = 0.53, *p* < 0.001) Internal consistency: Good; test-retest reliability of total weighted satisfaction (IoWS) at *r* = 0.72 (*p* < 0.001)
Fegg et al. (2016)	Palliative and Supportive Care	Schedule for Meaning in Life Evaluation (SMiLE)	English	Four major dimensions of meaning in life: leisure/health, work/finances, culture/spirituality, and relationships	*N* = 307; medical students Average age: 24.3	NA
George and Park (2017)	The Journal of Positive Psychology	Multidimensional Existential Meaning Scale (MEMS)	English	Comprehension Purpose Mattering	Sample 1: *N* = 188 Sample 2: *N* = 262 Sample 3: *N* = 160 Average age = 19	Reliability: Comprehension subscale: Cronbach's alpha = 0.90 Mattering subscale: Cronbach's alpha = 0.86
Hill et al. (2019)	Counselling Psychology Quarterly	Meaning in Life Measure (MILM)	English	Meaning in Life Measure (MILM) Two subscales: Experience (MILM-E) Reflectivity (MILM-R)	*N* = 401; American subjects	Internal consistency: Cronbach's alpha = 0.89 (MILM-E), 0.87 (MILM-R) Test-retest reliability: 0.82 (MILM-E), 0.79 (MILM-R)
Hutzell (1986)	The Hospice Journal	Life Purpose Questionnaire (LPQ)	English	Meaningfulness of life Purpose of life	*N* = 120	Test-retest reliability: High in the original study (*r* = 0.90); assessments with other populations are lacking
Li et al. (2021)	Journal of Happiness Studies	Quadripartite Existential Meaning Scale (QEMS)	English	Meaning in life (MIL): Feelings of coherence, purpose, and external value (significance or mattering) and internal value	Sample 1: *N* = 201, mean age = 19.9 Sample 2: *N* = 336, mean age = 20.3	Internal consistency: Comprehension: ω = 0.88, α = 0.87 Purpose: ω = 0.91, α = 0.91 Internal value (IV): ω = 0.91, α = 0.91 External value (EV): ω = 0.89, α = 0.88 Test-retest reliability: Correlations after 4 weeks: Comprehension = 0.65, Purpose = 0.74, IV = 0.72, EV = 0.74
Martela and Steger (2023)	The Journal of Positive Psychology	Three-Dimensional Meaning in Life Scale (3DM)	English	Meaning in Life: Significance Purpose Coherence	Study 1: *N* = 301; Study 2: *N* = 300; Study 3: *N* = 171; Study 4: *N* = 241; Study 5: *N* = 336 Age range = 19 to 71	Internal consistency: Coherence: Cronbach's alpha = 0.89 Purpose: Cronbach's alpha = 0.91 Significance: Cronbach's alpha = 0.89
Molina et al. (2023)	CES Psicología	Life Purpose Scale for Adolescents	Spanish	Search for Purpose in Life Identification of Purpose in Life	*N* = 554; mean age = 15.32 Argentina	Reliability: search component: Cronbach's alpha = 0.85 Identification component: Cronbach's alpha = 0.91 Overall: Cronbach's alphas ranging from 0.87 to 0.92 Search component: Explained 74.9% of the variance; Goodness of Fit Index (GFI) = 1.00
Reker and Peacock (1981)	Canadian Journal of Behavioural Science	Life Attitude Profile (LAP)	English	Life Purpose, Existential Vacuum, Life Control, Death Acceptance, Will to Meaning, Goal Seeking, Future Meaning to Fulfil	*N* = 219 students; mean age = 21.6	Higher-order factors: Account for 48% and 67% of total variance Internal consistency: Ranges from 0.83 (Life Purpose) to 0.55 (Future Meaning to Fulfil) Factor-to-composite correlations: Ranges from 0.34 (existential vacuum) to 0.62 (goal seeking), all significant at *p* < 0.001
Sahin and Derin (2023)	International Journal of Educational Research Review	Significance Quest Scale (SQS)	English	Quest for significance in Life	*N* = 621; age > 18 Turkish subjects	Internal consistency: Cronbach's alpha = 0.95 Convergent validity: 0.67
Salsman et al. (2020)	Quality of Life Research	Meaning and Purpose in Life (PROMIS)	English	Meaning and purpose, life satisfaction	*N* = 1,000; mean age = 47.8 Included a wide range of age groups	Internal consistency: 4-item Short Form: α = 0.90 37-item Bank: α = 0.98
Scheier et al. (2006)	Journal of Behavioral Medicine	Life Engagement Test (LET)	English	Purpose in life	*N* = 2,076 total in eight samples consisting of adults, students, and patients	Test-retest stability: Moderate, with correlations ranging from 0.61 to 0.76 Convergent validity: Correlates with various psychosocial measures and health-relevant variables
Schnell (2009)	Journal of Positive Psychology	Sources of Meaning and Meaning in Life questionnaire (SoMe)	English	Meaningfulness (5 items), Crisis of Meaning (5 items), and 26 sources of meaning grouped into four higher-order dimensions: Self-transcendence (vertical/horizontal), Self-actualization, Order, and Well-being/Relatedness	*N* = 616; range of age (16–85); Mean age = 45 German subjects	Internal consistency: α = 0.83–0.93 for the four higher-order dimensions; α = 0.65–0.95 for the 26 source scales (German representative sample, *N* = 603). Two-month test–retest coefficients average 0.81 for the scales and 0.90 for the dimensions; six-month stability 0.72/0.78 for sources and dimensions (crisis of meaning = 0.48). Extensive evidence of construct, content, discriminant, factorial, and incremental validity has been reported
Schnell and Danbolt (2023)	BMC Psychology	Meaning and Purpose Scales (MAPS)	English	Meaningfulness, Crisis of Meaning, and five “sources of purpose” scales: Sustainability, Faith, Security, Community, and Personal Growth	*N* = 974; range of age (18–89); mean age = 50 German subjects	Population-based German sample (*N* = 974); α = 0.74–0.96, ω = 0.75–0.96; all corrected item–total rs > 0.52; EFA/CFA support 2-factor (Meaningfulness, Crisis) and 5-factor (purpose) structure; test–retest ≥ 0.75 at 4 weeks and 2 months; convergent/divergent validity with SoMe, criterion validity (Sustainability with pro-environmental behavior), and predictive validity for general mental distress
Steger et al. (2006)	Journal of Counseling Psychology	Meaning in Life Questionnaire (MLQ)	English	Presence of meaning Search for meaning	*N* = 70; mean age = 20.1 Multiple ethnicities	Internal consistency: Presence of Meaning: α = 0.86 Search for Meaning: α = 0.88 for both Positive correlations: Life satisfaction, positive emotions, intrinsic religiosity, extraversion, agreeableness Negative correlations: Depression, negative emotions, neuroticism
Zambelli and Tagliabue (2024)	Journal of Happiness Studies	Situational Meaning in Life Evaluation (SMILE)	English	Comprehension Significance Purpose Presence and Search for meaning	Study 1 and Study 2: *N* = 3035 Mean age = 48.3 Study 3: *N* = 283; Mean age = 26 Italian subjects	Reliability: Presence of meaning: Ω = 0.84 Search for meaning: Ω = 0.83 Convergent Validity: CFI = 0.93

1. In Balthip et al. (2022), the column “Dimensions measured” incorrectly states “Meaning of life Self-worth Goal orientation.” The correct version is “Connectedness, meaning of life, self-worth, goal orientation, self-belief, determination, gratitude.”

2. In Li et al. (2021), the column “Dimensions measured” incorrectly states “Meaning in life (MIL): Feelings of coherence, purpose, and external value (significance or mattering).” The correct version is “Meaning in life (MIL): Feelings of coherence, purpose, and external value (significance or mattering) and internal value.”

3. In Reker and Peacock (1981), the column “Dimensions measured” incorrectly states “Life purpose, Existential vacuum Will to meaning, Goal seeking”. The correct version is “Life Purpose, Existential Vacuum, Life Control, Death Acceptance, Will to Meaning, Goal Seeking, Future Meaning to Fulfil.”

4. In Schnell (2009), the column “Scale name” incorrectly states “Sources of Meaning (SoMe) scale.” The correct version is “Sources of Meaning and Meaning in Life questionnaire (SoMe).”

5. In Schnell (2009), the column “Dimensions measured” incorrectly lists “Meaningfulness Crisis of meaning.” The correct version is “Meaningfulness (5 items), Crisis of Meaning (5 items), and 26 sources of meaning grouped into four higher-order dimensions: Self-transcendence (vertical/horizontal), Self-actualization, Order, and Well-being/Relatedness.”

6. In Schnell (2009), the column “Psychometric properties” incorrectly lists “Meaningfulness: α = 0.74 Crisis of Meaning: α = 0.92.” The correct version is “Internal consistency: α = 0.83–0.93 for the four higher-order dimensions; α = 0.65–0.95 for the 26 source scales (German representative sample, *N* = 603). Two-month test–retest coefficients average 0.81 for the scales and 0.90 for the dimensions; six-month stability 0.72/0.78 for sources and dimensions (crisis of meaning = 0.48). Extensive evidence of construct, content, discriminant, factorial, and incremental validity has been reported.”

7. In Schnell and Danbolt (2023), the column “Dimensions measured” incorrectly appears as “Meaningfulness, Crisis of Meaning Sustainability, Faith, Security Community, Personal Growth.” The correct version is “Meaningfulness, Crisis of Meaning, and five “sources of purpose” scales: Sustainability, Faith, Security, Community, and Personal Growth.”

8. In Schnell and Danbolt (2023), the column “Psychometric properties” incorrectly states “Internal Consistency: α = 0.70, Ω = 0.71 Correlation with MAPS Sustainability Scale: *r* = 0.44 (*p* = 0.001).” Correlation with Other MAPS Scales: Personal Growth: *r* = 0.06 (ns). The correct version is “Population-based German sample (*N* = 974); α = 0.74–0.96, ω = 0.75–0.96; all corrected item–total rs > 0.52; EFA/CFA support 2-factor (Meaningfulness, Crisis) and 5-factor (purpose) structure; test–retest ≥ 0.75 at 4 weeks and 2 months; convergent/divergent validity with SoMe, criterion validity (Sustainability with pro-environmental behaviour), and predictive validity for general mental distress.”

The corrected Table 1 appears below.

There was a mistake in Table 2 as published.

**Table 2:** Summary of study quality assessments performed using the Downs and Black and STROBE checklists.

Study	Quality assessment tool						
	Downs and black checklist						
	Overall quality max score = 11	External validity max score = 3	Internal validity bias max score = 7	Internal validity confusion max score = 6	Power max score = 5		Score out of 32
Balthip et al. (2022)	7	3	5	6	5		26
Bellet et al. (2019)	10	2	3	1	5		21
Campbell and Baumeister (1973)	7	2	3	1	2		15
Caycho-Rodríguez et al. (2023)	9	2	4	3	5		23
Chang and Dodder (1984)	5	2	3	0	3		13
Fegg et al. (2008)	7	2	4	2	4		19
George and Park (2017)	9	3	5	6	5		28
Hill et al. (2019)	6	3	2	0	4		15
Hutzell (1986)	7	0	2	0	3		12
Li et al. (2021)	7	2	2	2	3		16
Martela and Steger (2023)	8	2	2	1	5		18
Molina et al. (2023)	7	3	3	2	2		17
Reker and Peacock (1981)	9	3	6	4	4		26
Sahin and Derin (2023)	8	0	3	0	4		15
Salsman et al. (2020)	7	3	3	0	4		17
Scheier et al. (2006)	9	2	2	1	4		18
Schnell (2009)	9	2	3	1	5		20
Schnell and Danbolt (2023)	8	**3**	3	0	**5**		**19**
Steger et al. (2006)	9	2	3	1	5		20
Zambelli and Tagliabue (2024)	7	3	3	0	4		17
Study	STROBE checklist						
	Title and summary max score = 1	Introduction max score = 2	Methods max score = 9	Results max score = 5	Discussion max score = 4	Other information max score = 1	Score out of 22
Chamberlain and Zika (1988)	1	2	8	4	4	0	19
Crumbaugh (1977)	1	2	7	5	3	0	18
Fegg et al. (2016)	1	2	7	3	3	0	16

1. In Schnell and Danbolt (2023), the column “External validity max score = 3” incorrectly shows “0.” The correct version is “3.”

2. In Schnell and Danbolt (2023), the column “Power max score = 5” incorrectly shows “3.” The correct version is “5.”

3. In Schnell and Danbolt (2023), the column “Score out of 32” incorrectly shows “14.” The correct version is “19.”

The corrected Table 2 appears below.

In section **3. Results, 3.2 Sample characteristics** a paragraph should be added after the sentence “Other tools, such as the Meaning in Life Questionnaire (MLQ) published by Steger et al. (2006), are widely used across different demographic groups, demonstrating broad applicability.”

The full paragraph is below:

“The Meaning and Purpose Scales (MAPS; Schnell and Danbolt, 2023) were validated in several large German samples, including a development study with *N* = 13,660 participants and a population-based sample of adults (*N* = 974). The authors report good to excellent internal consistency for all scales (Cronbach's α = 0.74–0.96; McDonald's ω = 0.75–0.96), corrected item–total correlations above 0.52, and stable two- and five-factor structures for meaning and sources-of-purpose scales confirmed by CFA across gender and age groups. Test–retest reliability over 4 weeks and 2 months was at least 0.75 for every scale, and extensive evidence of convergent and divergent validity with the SoMe, criterion validity (e.g., Sustainability predicting pro-environmental behaviour), and predictive validity for general mental distress was provided.”

In **Section 3. Results, 3.2 Sample characteristics**, in the following sentence “For instance, while the MLQ has been validated in multiple cultural contexts, its applicability to non-Western populations remains limited due to its emphasis on individualistic notions of meaning and purpose. Psychometric Properties.” **“Psychometric Properties**” should appear as a separate heading and not an isolated phrase.

In section **3. Results, 3.2 Sample characteristics**, the following paragraph should be modified: “To ensure the quality and reliability of the included studies, we employed two well-established checklists for quality assessment: the Downs and Black checklist and the STROBE checklist. The Downs and Black checklist were used to assess the methodological quality of the 20 experimental studies that were included in our review. The total scores obtained for these studies ranged from 12 to 28. Thirteen studies were categorized as high quality (≥75% of the maximum score), studies with higher quality scores were given greater weight in the synthesis of findings, as they were deemed more methodologically robust and reliable. For the three non-experimental studies, namely Chamberlain and Zika (1988), Crumbaugh (1977), and Fegg et al. (2016), we utilized the STROBE checklist and found that the total scores ranged from 16 to 19. The detailed outcomes of the quality assessments for the experimental studies, conducted using the Downs and Black checklist, are summarized in Table 2. This table provides a clear and structured evaluation of the methodological quality of these studies. This dual assessment approach ensures a rigorous and systematic evaluation of the included studies, thereby enhancing the credibility and reliability of our systematic review findings.”

The correct paragraph is below:

“To appraise the methodological quality of the included studies, we used two complementary checklists. First, the Downs and Black Quality Index was applied to the 20 empirical studies that reported quantitative analyses of associations or group differences, regardless of whether their design was strictly experimental or non-experimental. This instrument was originally developed to assess the methodological quality of randomized and non-randomized intervention studies, but most of its items refer to generic aspects of study quality (e.g., clarity of hypotheses and outcomes, description of participants, use of appropriate statistics). In non-experimental and psychometric validation studies, items that referred specifically to random allocation, blinding or follow-up were difficult to apply and were scored conservatively as 0, which likely leads to an underestimation of certain aspects of methodological rigour in these designs. Second, for the three studies that were purely observational and descriptive (Chamberlain and Zika, 1988; Crumbaugh, 1977; Fegg et al., 2016), we did not use the Downs and Black Index and instead assessed reporting quality using the STROBE checklist. In our synthesis, higher Downs and Black scores were interpreted as indicating better overall methodological quality among the analytical studies, whereas higher STROBE adherence reflected more comprehensive and transparent reporting in the descriptive observational studies.”

The original version of this article has been updated.

